# Chronic Functional Constipation and Encopresis in Children in Relationship with the Psychosocial Environment

**DOI:** 10.1155/2016/7828576

**Published:** 2016-11-21

**Authors:** Claudia Olaru, Smaranda Diaconescu, Laura Trandafir, Nicoleta Gimiga, Radian A. Olaru, Gabriela Stefanescu, Gabriela Ciubotariu, Marin Burlea, Magdalena Iorga

**Affiliations:** ^1^“Gr. T. Popa” University of Medicine and Pharmacy, Iasi, Romania; ^2^“Sf. Maria” Emergency Hospital for Children, Iasi, Romania; ^3^“Socola” Emergency Hospital, Iasi, Romania; ^4^“Sf. Spiridon” Emergency Hospital, Iasi, Romania

## Abstract

Functional constipation is an issue for both the patient and his/her family, affecting the patient's psychoemotional balance, social relations, and their harmonious integration in the school environment. We aimed to highlight the connection between chronic constipation and encopresis and the patient's psychosocial and family-related situation.* Material and Method*. 57 patients with ages spanning from 6 to 15 were assessed within the pediatric gastroenterology ward. Sociodemographic, medical, and psychological data was recorded. The collected data was processed using the SPSS 20 software.* Results*. The study group consisted of 57 children diagnosed with encopresis (43 boys (75.44%) and 14 girls (24.56%)), *M* = 10.82 years. It was determined that most of the children came from urban families with a poor socioeducational status. We identified a level of studies of 11.23 ± 5.56 years in mothers, while fathers had an average number of 9.35 ± 4.53 years of study. We also found a complex relationship between encopretic episodes and school performances (*F* = 7.968, *p* = 0.001, 95% Cl). Children with encopresis were found to have more anxiety/depression symptoms, greater social problems, more disruptive behavior, and poorer school performance.* Conclusions*. The study highlights the importance of the family environment and socioeconomic factors in manifestations of chronic constipation and encopresis.

## 1. Introduction

Functional constipation is characterized by infrequent stool evacuation, passing of hard stools, or painful defecation with no fundamental organic cause [1]. Up to 84% of the children with chronic constipation experience frequent episodes of fecal incontinence [2]. Chronic constipation and secondary fecal incontinence are a source of concern for the child and his/her family. The symptoms are often persistent and relapses are frequent [3–5]. Fecal incontinence can also cause feelings of guilt and discomfort and is associated with social withdrawal behaviors, anxiety, and depression [6–8]. Encopresis is defined as a disorder characterized by repeated stool evacuation in inappropriate places in children over the age of four. The behavior can be either involuntary or intentional; it must be present for a minimum of three months at a rate of at least once a month and is not the direct effect of a substance or a medical condition [9]. Biological and developmental mechanisms can be responsible in the etiology of encopresis, and so can psychosocial and environmental factors. Many of the children presenting with encopresis have experienced a previous event that triggered the disorder by making the bowel movement uncomfortable or scary [10]. Such an event can vary from constipation associated with painful bowel movement or fear of using the toilet to repeated sexual abuse. The prevalence of encopresis was assessed in around 1–3% of the general pediatric population [11, 12]. Reports show that this rate is higher (4%) in developing countries [13]. No encopresis-related studies were carried out on the pediatric population in Romania [14, 15]. The long-term results and factors influencing the prognosis are debatable: while some studies reported behavior disorders and the family environment as predictors of poor outcomes in nonretentive encopresis, Montgomery and Navarro [16] and Van Wering et al. [17] described retentive encopresis as being negatively correlated with the favorable evolution and risk factors could not be determined. The aim of this study was to highlight the sociodemographic characteristics of the encopretic patients and those of their families and the occurrence of behavioral issues, as well as identify the depressive and anxious disorders occurring within this group. For this purpose there was a focus on identifying the social and family-related environment conditions by determining the level of education and current profession of the child's next of kin, studying the changes in terms of somatization and behavior in patients struggling with constipation, and establishing some correlations between the severity of clinical aspects and the psychosocial impact on both the patient and his/her family.

## 2. Material and Method

The prospective cohort study was carried out on a group of 57 patients and spanned on a period of 20 weeks. The study included children aged 6 to 15 that were admitted to the gastroenterology unit of a tertiary hospital from north-eastern Romania. The study included underage patients and their next of kin, who were informed in advance with regard to the purposes of the study and signed an informed consent form prior to the inclusion. The inclusion criteria are as follows: patients with at least one encopretic episode per week, spanning over at least one year. The exclusion criteria are as follows: documented mental retardation or any neuromuscular or gastrointestinal disorders associated with organic constipation, attention deficit/hyperactivity, or obsessive-compulsive disorders. Of all the 67 patients that were diagnosed with encopresis, 8 were excluded due to the parents' refusal to participate in the study and 2 abandoned the study during the assessment phase, with the final group including a total of 57 patients. The medical history phase included gathering information regarding the parents' level of education and their level of professional certification. We also recorded school-related data: education and training level, school results, absenteeism, and abandonment. For the purpose of assessing the clinical symptoms of encopresis, children and their parents were required to record the frequency of such symptoms in a diary for a month. Laxative-based treatment was stopped during this month. To determine the impact of encopretic disorders on the psychoemotional balance, all patients underwent psychological examination by a clinical psychologist through observation, structured interview, the ASEBA scales [18], and projective tests. ASEBA scales were used to describe the child's behavior. An assessment can quickly and effectively assess diverse aspects of adaptive and maladaptive functioning (schizoid or anxious, depressed, uncommunicative, obsessive-compulsive, somatic complaints, social withdrawal, hyperactive, aggressive, delinquent, sex problems, etc.). Interview and projective tests were used to identify the personal beliefs and the impact of the disease on daily life. Results were used also to build the individual therapy.

## 3. Results

**Figure 1:**
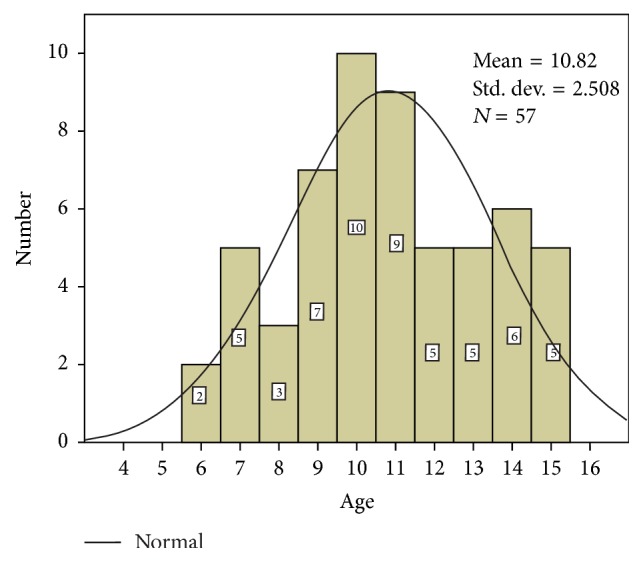
Distribution of patients according to the age.

A total of 57 underage patients participated in the study. The demographic characteristics and medical data are described in Table 1. Children were aged between 6 and 15 (median age 10.82 ± 2.507) (Figure 1).

Of all the subjects, 75.44% (*N* = 43) were males and 24.56% (*N* = 14) were females. The M : F ratio is 3.07 : 1. As far as the origin community is concerned, 59.65% came from urban areas and 40.35% came from rural areas (Table 1).

68.42% (39/57) of children received treatment with oral laxatives 6 months before the enrollment. Rectal enemas were used at the beginning of the treatment as disimpaction therapy; 19.29% (11/57) of children were treated with oral laxatives and underwent dietary changes (fiber rich diet and toilet training), and 12.28% (7/57) underwent dietary changes. Only 7.01 (4/57) of the children went to a psychologist. All of them were from urban areas. 12.28% (7/57) declared that they did not follow the recommended treatment. 85.71% (6/7) were from rural areas. The frequency of encopretic episodes ranged between 18 episodes/month and 41 episodes/month, with an average of 28.3 ± 6.67.

As far as the social and family environment is concerned, 14.03% (8/57) of the children were living in single parent families, 19.29% (11/57) of the children had one parent working abroad, and 7.01% (4/57) had both parents working abroad and lived with their grandparents.

The study considered variables related to the parents' level of education, profession, and their addressability to medical services (the time from the onset of the symptoms to first medical consult). In terms of educational level, we noted that 50% of the parents (*N* = 57) had finished middle school (8 grades) and 15.78% (*N* = 18) completed professional studies, while 34.22% (*N* = 39) of the participants had completed secondary or higher education (high school and university diplomas). Another variable we tracked was the number of years of study averaged by the parents. Thus we identified a level of studies of 11.23 ± 5.56 years in mothers, while fathers had an average number of 9.35 ± 4.53 years of study. The analysis of current occupation and professional status showed that the parents included in the study worked in various fields: 48 workers, 23 intellectuals, 28 unemployed persons, and 15 people who retired for medical reasons. The ANOVA test interpretation revealed that the number of encopretic episodes per month was influenced by the level of education of the patients' female next of kin. (*F* = 2.684, *p* = 0.008, 95% Cl) (Table 2).

**Figure 2:**
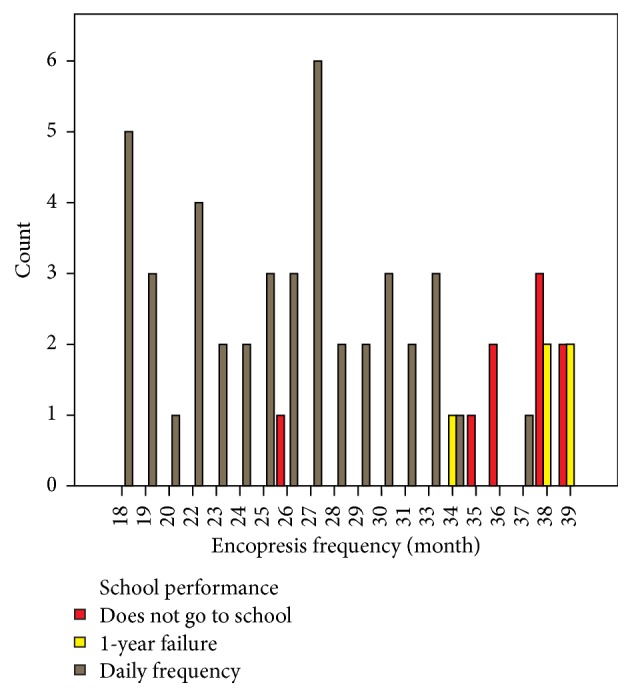
Distribution of patients according to school performance and number of encopretic episodes per month.

Education-related data was collected during interviews with the next of kin and conversations with the patient. We quantified the level of education and number of missed school days within the studied group. Of the 57 children included in the group, 9 (15.78%) had abandoned school and 5 (8.77%) failed one year of study (Figure 2).

**Figure 3:**
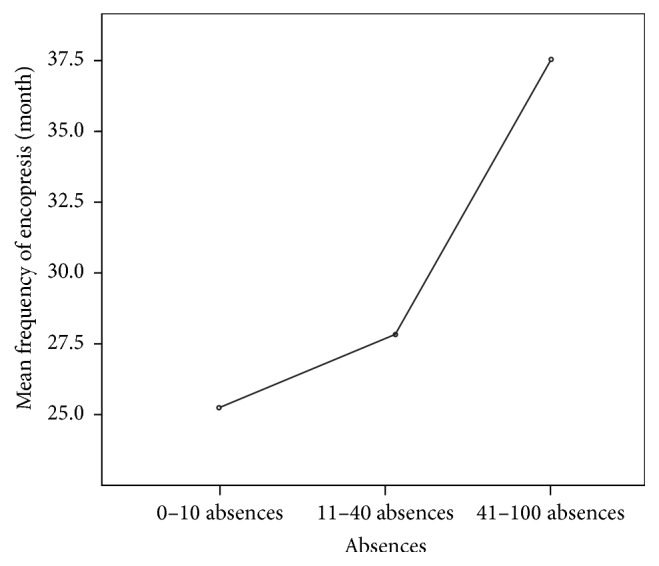
The average value of encopretic episodes' frequencies compared to the number of missed classes.

21 of the children had around 0–10 missed classes, 31 children had around 11–40 missed classes, and 5 of them had a large number of missed classes, namely, around 41 and 100 per semester. The ANOVA test interpretation revealed that the number of encopretic episodes per month influences the number of missed classes. (*F* = 7.968, *p* = 0.001, 95% Cl) (Table 3), (Figure 3).

Psychological data were collected in order to shape a psychological profile for children with encopretic and constipation problems. The psychological evaluation identified (in various associations) psychomotor agitation (*N* = 9; 15.79%), anxiety (*N* = 22; 38.59%), affective deprivation (*N* = 30; 52.63%), social adjustment difficulties (*N* = 13; 22.81%), introversion (*N* = 12; 21.05%), low frustration tolerance (*N* = 11; 19.29%), depressive syndrome (*N* = 8; 14.03%), speech disorders (*N* = 5; 8.76%), and emotional distress (*N* = 19; 33.31%) (Table 4).

## 4. Discussions

The average age in our group of patients with encopresis was 10.82 years. The data resulting from this study were different from other data in the literature, which indicate a higher prevalence in small children. A population-based study conducted in the Netherlands which involved 13,111 parents and their 5- to 6-year-old children and 9,780 parents and their 11- to 12-year-old children revealed that the prevalence of encopresis was 4.1% in the 5-to-6 age group and 1.6% in the 11-to-12 age group. Encopresis was more frequent among boys and children from the poorest areas of the city [19]. Similar results were also discovered in the population of southeast Nigeria. The authors of the study showed that encopresis affected 3% of 4-year-old and 1.6% of 10-year-old children. It occurs more commonly in the 5- to 10-year-old group and less frequently in adolescence, and it predominantly affects males [20]. Encopresis also occurs in adolescents and even among adults; however, the prevalence is unknown in those age groups [21]. As far as gender distribution is concerned, our results are consistent with the studies of the authors mentioned above [19, 20]. The patients' geographic area of origin was predominantly urban. The low frequency of patients from rural areas in our study could be a result of delayed diagnosis due to reduced access to medical services in some disadvantaged communities, as well as the ignorance of the symptomatology by the patients' next of kin with a lower level of education. This idea is also supported by the fact that the average time lapsed from the onset of the disorder to the patients' seeing a doctor was longer in rural areas compared to urban areas (11.7 weeks/7.8 weeks). A highlight in our study is that only 59.7% of the patients lived with both parents. Children from broken homes can present a higher risk of developing emotional and behavioral disorders. Literature data confirms that the structure of the family into which a child develops entails some disadvantages that subsequently affect cognitive, socioemotional, and even physical health outcomes. For example, high cognitive scores and less socioemotional or health disturbances were registered in children with married parents [22–24]. Time allocated to raising and caring for children is expected to be positively correlated with their wellbeing [25]. While the quality of the time a parent spends with the child is important, studies have shown that quantity of such time also has positive consequences for child cognition and health [26]. This research points specifically to the likely negative effects of paternal absence, results proved by other studies that identified that the absence of either mother or father has great impact on children's development [22, 27]. Changes in family structure are typically accompanied by changes in economic, time, and parental resources; these in turn place stress on families and thus adversely affect child outcomes. Family instability also yields residential instability and a sense of insecurity concerning household rules, leading to an increased rate of behavioral problems, low rate of cognitive achievements, and poorer health [22, 28–30]. In our study, most of the parents (65.78%) of children with encopresis had a low educational level. The medical history analysis highlighted that most of the children that did not observe the previous prescribed courses of treatment came from rural areas and their parents had a low level of education (85.71%), while all the children that had seen a psychologist were from urban areas and their parents had completed secondary or higher education. Considering that the treatment for encopresis is based particularly on the close observance of therapeutic indications [31, 32], we can speculate that parents with a higher level of education might be more compliant. Parental education was reported both as a competence marker for toilet training and as a factor of protection against the stress of living in an underprivileged family [33]. In our study, children whose mothers had a high educational level reported a lower number of encopretic episodes per month (*F* = 2.684, *p* = 0.008, and 95% Cl). An important question is whether the frequency of encopretic episodes influences school performances. The analysis on the rate of enrollment, school absenteeism, and the children's capacity to complete the academic year showed that children with encopresis have learning disabilities and poor school performances and miss school days more frequently. Lower rates were registered for scales measuring spelling and arithmetic skills using Wide Range Achievement Test (WRAT), as Stern et al. identified [34, 35]. The relationship between health status and academic achievement is more complex than it would seem at first glance. While there is strong evidence that children whose healthcare needs are met are less likely to miss school days because of illness, school performance is multidetermined. These risk factors include parental attitudes and beliefs, patterns of mother-child interaction, maternal education, socioeconomic status, family social support, family size, stressful life events, and the child's cognitive functioning. It was proved that there are psychosocial factors which affect academic outcomes as well as emotional development. Children exposed to these factors are at heightened risk for emotional and behavioral problems and school failure [36]. Children in homeless families experience a high rate of academic failure consistent with the need for special education evaluation and services [37].

Encopretic children are a particularly vulnerable social group, being exposed to social risks in terms of losing their sense of belonging both to their own generation group and the entire society, reaching a marginal position in society. Stigmatized by parents, peers, neighbors, and society, encopretic patients have fragile personalities that need tolerance, intercommunication, and a lot of trust from other people. Several authors have reported finding poorer self-esteem in children with encopresis. Low scores regarding self-esteem were identified by Landman et al. in children with encopresis, comparing to a control group formed by children with chronic physical problems [34, 38]. Lower self-esteem in encopretic children than in nonsymptomatic ones was also reported by Owens-Stively [34, 39]. The most frequent changes encountered in our study included emotional distress, anxiety, and social adjustment difficulties. There was a high rate of somatization and behavioral disorders in our group and their composition was largely heterogeneous. Literature data shows that the association of encopresis with behavioral disorders has led to an unfavorable prognosis of this disorder [40, 41]. Levine et al. found that children with encopresis who did not respond to treatment scored higher on antisocial-aggressive behaviors before treatment [42]. They also reported differences between a nonsymptomatic comparison group and the children with encopresis prior to treatment on affective-dependent behavior (i.e., those who demonstrated signs of anxiety and depression). In a study by Johnston and Wright, attention deficit or hyperactivity was identified for 23% of cases of encopretic children [43]. A number of studies have demonstrated poorer social skills and higher withdrawal behavior in children with encopresis [42, 44].

Some authors believe that, in predisposing to and perpetuating encopresis, the approach of toilet training, not the time of its initiation, seems to be the factor that matters [45]. Regarding the psychological treatment, attention and behavior problems may be the target. Treatment of these problems may increase treatment compliance and prevent conflicts that may occur within the family in relation to these problems because a lot of studies pointed the impact of child behavior related to family conflicts [46–48]. On the other hand, familial factors such as maternal depression and/or anxiety symptoms are associated with elimination disorders at school age and including factors related to family functioning must be included in the psychological intervention [49].

Our study has some limitations. This is a single center report and is limited to children addressed to our gastroenterology center. It is not clear whether the results would hold for a nationally representative sample of children in Romania. Because the period covered by the study was also relatively short, it is not clear whether the disparities between children of parents with more or less education remain as children age and how they affect a larger set of outcomes.

Our analysis leaves many questions for future research. The behavioral disorders noticed in children with encopresis could be either a result of their excessive concern with uncontrollable encopretic accidents and the resulting social tension or a result of some developmental delays that could ultimately play a part in the development or persistence of encopresis. Another critical question is the extent to which specific policies and programs dedicated to children can help address the observed deficits (improvement of access to health care services and various means of cognitive development). Future research is necessary in this direction.

## 5. Conclusions

The findings of this study allow us to outline a profile of encopretic patients with respect to some of the psychosocial factors involved in the multifactorial determinism of this disorder. Educating parents on the association with somatization and behavioral disorders can lead to a more effective diagnosis and a better response to treatment for children with constipation and fecal incontinence. Screening for behavioral disorders in encopretic patients could be useful for their therapeutic management. A more aggressive treatment for constipation can be justified in these patients. In the cases that associate severe behavioral disorders, early diagnosis and multidisciplinary therapeutic approach can be useful for both the child and families.

## Figures and Tables

**Table 1:** Demographic characteristics, history, and clinical features of the patient series.

*Demographics*		
Age (median)	10.82	(6–15)
Sex (M) (*N*/%)	43/57	75.44%
Area of origin (urban) (*N*/%)	34/57	59.65%
*History*		
Family history of functional constipation (*N*/%)	8/57	14.03%
Time lapsed from the onset of symptoms (week) to the first medical consult (urban/rural)	7.8/11.7	(1–17)/(5–19)
Duration of symptomatology (years)	4.21	(2–7)
Average duration of treatment (months)	14	(6–32)
*Symptoms*		
Frequency of defecation/week	1.32	(1–3)
Encopretic episodes/month	28.3	(18–41)
Urinary incontinence (*N*/%)	9/57	15.78%
Abdominal pain (*N*/%)	34/57	59.64%
Stool passing pain (*N*/%)	46/57	80.70%

**Table 2:** Encopresis frequency and relation with the level of education of the patients' female next of kin.

ANOVA frequency of encopresis/month					
	Sum of squares	df	Mean square	*F*	Sig.
Between groups	1055.652	12	87.971	2.684	.008
Within groups	1442.278	44	32.779		
Total	2497.930	56			

**Table 3:** Frequency of encopretic episodes and relationship with the school absenteeism.

ANOVA frequency of encopresis/month					
	Sum of squares	df	Mean square	*F*	Sig.
Between groups	569.178	2	284.589	7.968	.001
Within groups	1928.751	54	35.718		
Total	2497.930	56			

**Table 4:** Psychological evaluation.

Psychological examination	Number of cases	Percentage
Psychomotor agitation	9	15.78%
Anxiety	22	38.59%
Panic attack	1	1.75%
Tic disorder	2	3.5%
Affective deprivation	30	52.63%
Social adjustment difficulties	13	22.81%
Low average IQ	2	3.51%
Negativism	7	12.28%
Irritability, irascibility	6	10.52%
Acute reaction to stress	1	1.75%
Depressive syndrome	8	14.03%
Shyness	12	21.05%
Low tolerance to frustration	11	19.29%
Speech disorders	5	8.76%
Emotional distress	5	8.76%
Hypochondriac tendencies	1	1.75%
